# Assessment of Occupational Exposure to Airborne Phenol and Biological Monitoring of Accumulation Trends

**DOI:** 10.3390/healthcare13131516

**Published:** 2025-06-25

**Authors:** Gyu-Jin Sim, Sun-Haeng Choi, Ki-Youn Kim

**Affiliations:** 1 Graduate School of Safety Engineering, Seoul National University of Science and Technology, Seoul 01811, Republic of Korea; 2 Department of Occupational and Environmental Medicine, College of Medicine, Chungbuk National University, Cheongju 28644, Republic of Korea; viopxo@chungbuk.ac.kr; 3 Department of Safety Engineering, Seoul National University of Science and Technology, Seoul 01811, Republic of Korea

**Keywords:** airborne phenol, chemical industry, occupational health, personal protective equipment, phenol exposure, smoking, urinary phenol

## Abstract

**Introduction:** This study investigates the relationship between occupational exposure to airborne phenol and its biological accumulation in chemical plant workers, with a particular focus on urinary phenol levels. It also explores the influence of job roles, employment duration, protective equipment use, and personal lifestyle habits on internal exposure. **Methods:** A cohort of bisphenol A (BPA) manufacturing workers was classified based on job tasks and exposure characteristics. Airborne phenol concentrations were measured using standard occupational hygiene methods, and urinary phenol levels were analyzed before and after work shifts. Statistical analyses examined associations between phenol exposure and occupational and behavioral variables. **Results:** Urinary phenol levels significantly increased after work shifts, particularly among workers involved in cleaning tasks. A strong correlation was observed between airborne phenol concentrations and urinary levels, indicating that even low-level environmental exposure can result in measurable biological accumulation. Notably, workers who did not use personal protective equipment or who reported smoking showed higher internal phenol burdens. **Conclusions:** This study highlights the importance of integrating biological monitoring with conventional exposure assessments in phenol-handling workplaces. Effective exposure control should include improved ventilation, strict compliance with personal protective equipment use, and health education programs that address modifiable lifestyle factors such as smoking. These findings underscore the need for comprehensive strategies to reduce occupational health risks associated with phenol exposure.

## 1. Introduction

Phenol (C6H5OH) is an aromatic compound where a hydroxyl group (-OH) is attached to a benzene ring. It is a key chemical widely utilized in modern industrial society, serving as a primary raw material in the manufacturing of plastics, pharmaceuticals, disinfectants, and cosmetics [1,2]. Additionally, phenol is included as a component in local anesthetics and preservatives. However, due to its high toxicity and rapid absorption in the human body, systematic workplace monitoring and scientific evaluation of occupational exposure are required [3]. Phenol is absorbed primarily through the respiratory tract, skin, and digestive system, with inhalation exposure reported to have an absorption rate of 70–80% [4].

While acute exposure to high concentrations of phenol can cause severe local and systemic effects, including chemical burns, respiratory distress, and central nervous system depression, chronic low-level exposure, as often encountered in occupational settings, poses a more insidious threat. Long-term exposure to phenol has been associated with potential clinical implications such as renal dysfunction, hepatic damage, and neurological disturbances [5]. Therefore, understanding the accumulation trends and identifying contributing factors is clinically significant for early detection, prevention, and management of these potential health issues among exposed populations.

Additionally, phenol’s skin absorption rate measures approximately 10.9%, suggesting that skin contact represents a significant exposure pathway [6,7]. Once absorbed, phenol rapidly distributes throughout the body via the bloodstream [8], accumulating at relatively high concentrations in the liver, spleen, kidneys, adrenal glands, and lungs [6].

Phenol metabolism predominantly occurs in the liver, where it undergoes conjugation with glucuronic acid to form phenyl glucuronide or is converted to phenyl sulfate through sulfation [9]. As phenol concentrations increase, a metabolic shift from sulfation to glucuronidation is observed, primarily due to the limited availability of 3-phosphoadenosine-5-phosphosulfate (PAPS), which is required for sulfation. These metabolites, once synthesized in the liver, are excreted through either bile or the bloodstream, with the majority ultimately eliminated via urinary excretion [10].

Inhalation of vapor in the workplace is recognized as one of the primary routes of phenol exposure. Grazyna [11] reported that workers in coke production facilities exposed to 4.50 mg/m^3^ of airborne phenol exhibited significantly higher urinary phenol levels compared to non-exposed individuals. Similarly, a U.S. cohort study involving BPA plant employees found urinary phenol/BPA concentrations to be up to 70-fold higher than those observed in the general population [12].

Internationally, the American Conference of Governmental Industrial Hygienists (ACGIH) has established a Threshold Limit Value–Time Weighted Average (TLV-TWA) of 5 ppm for phenol based on an 8 h workday, which has been equally adopted in South Korea [13]. To clarify the conversion, 1 ppm of phenol at 25 °C and 760 mmHg is approximately equivalent to 3.85 mg/m^3^. Therefore, a TLV-TWA of 5 ppm corresponds to approximately 19.25 mg/m^3^. Additionally, the Biological Exposure Index (BEI) for occupational exposure assessment is set at 250 mg/g creatinine, serving as a crucial standard for exposure monitoring and health risk evaluation.

Phenol exposure can occur through various pathways outside the workplace environment. Tobacco smoke notably contains significant amounts of phenol, with reported levels of 60–140 μg in non-filter cigarettes, 19–35 μg in filter cigarettes, and 24–107 μg in cigars [5,14,15,16,17,18,19,20,21,22,23]. These findings suggest that environmental factors also significantly impact phenol accumulation in the body, emphasizing the need for more comprehensive exposure assessments. While previous studies have investigated occupational phenol exposure [22], few have comprehensively explored the combined impact of low-level airborne phenol exposure, various occupational factors (e.g., job role, working hours, PPE usage), and personal lifestyle habits (e.g., smoking, alcohol consumption) on biological accumulation. This study aims to fill this gap by providing a more holistic understanding of phenol exposure in real-world industrial settings.

Based on this background, the present study aims to measure urinary phenol concentrations in workers exposed to low-level airborne phenol and to analyze the effects of occupational and lifestyle factors on phenol absorption. Through this study, we seek to provide scientific evidence and practical guidelines for mitigating occupational phenol exposure in workplace environments. Specifically, this study addresses the following research questions:

- What is the correlation between airborne phenol concentration and urinary phenol levels in chemical plant workers?
- How do job role, duration of employment, and personal protective equipment usage affect urinary phenol accumulation?
- What is the impact of personal lifestyle habits, such as smoking and alcohol consumption, on urinary phenol levels?

## 2. Materials and Methods

### 2.1. Study Subjects

This study was conducted on 41 workers employed at a bisphenol A (BPA) manufacturing facility located in Yeosu, South Korea. The facility was selected as the study site because it includes specific production processes involving the handling of phenol, providing an appropriate setting for evaluating occupational exposure. The study subjects were limited to workers who directly handled phenol to ensure the reliability of exposure assessment. To enhance the validity of the study, only those with a minimum period of work experience in a consistent occupational environment were selected.

The mean age of participants was 40.7 ± 6.4 years, with an average total employment duration of 15.3 ± 1.6 years and an average duration in the current job of 4.1 ± 2.7 years. All participants were informed about the study objectives and procedures and provided written informed consent. The study protocol was approved by the Institutional Review Board (IRB No. 2023-0035-02) of the Seoul National University of Science and Technology.

The urine samples were collected before and after the work shift on 18 April 2023.

### 2.2. Research Methods

#### 2.2.1. Questionnaire Survey

A standardized, self-administered questionnaire was used to evaluate the general characteristics and occupational exposure factors of the study participants. The questionnaire was distributed and collected in advance from 11 to 15 April 2023. This questionnaire was structured according to the research objectives and included demographic information (age, total years of employment, years in current process, working hours), job-related factors (process assignment), and lifestyle habits (alcohol consumption and smoking status).

The questionnaire was developed based on existing validated tools for occupational health surveys and was adapted to specifically address phenol exposure in the workplace. A pilot study was conducted with a small group of workers (n = 5) to ensure clarity and comprehensibility of the questions before the main survey. Specific variables related to phenol exposure included questions on the frequency and duration of direct contact with phenol-containing materials, the use of personal protective equipment (PPE), and awareness of safety protocols.

#### 2.2.2. Work Classification

Work classifications were categorized into three groups based on the level of phenol exposure. The characteristics of each work type are detailed in Table 1.

#### 2.2.3. Measurement and Analysis of Airborne Phenol

The measurement of airborne phenol concentrations in the work environment was conducted in accordance with the *NIOSH Manual of Analytical Methods* (NIOSH Method No. 2546) [15]. Personal air sampling was performed on 41 study subjects using low-flow personal air samplers (Gilian low-flow pump, Irvine, CA, USA), which were attached to each worker. Air samples were collected for a minimum of 6 h using XAD-7 adsorption tubes (100 mg/50 mg) at a flow rate of 0.1 L/min.

The analysis of airborne phenol was conducted using high-performance liquid chromatography (HPLC) under the following analytical conditions (Table 2).

#### 2.2.4. Measurement and Analysis of Urinary Phenol

Pre- and post-shift urine samples were collected to determine urinary phenol concentrations. The collected samples were stored at −20 °C until transported to the laboratory for analysis. Urinary samples were collected before and after the work shift on 18 April 2023. Each sample was stored in a sealed polyethylene container at a temperature below 4 °C and transported using an icebox to ensure the integrity of the samples during transportation. The sample handling and transportation procedures followed standardized guidelines as specified in the standard methods for biological specimen testing and were consistent with the industrial hygiene testing protocols described by LabCorp (2023) [24]. This protocol recommends sampling at the end of the shift and refrigerated storage for up to 3 days (below 4 °C). Additionally, according to the toxicological profile of phenol by ATSDR (2008), phenol in urine remains stable for up to 4 days under refrigerated conditions and up to 3 months under frozen conditions [5]. All collected samples were analyzed on 10 May 2023, at the Industrial Hygiene Engineering Laboratory of Seoul University of Science and Technology.

**Figure 1:**
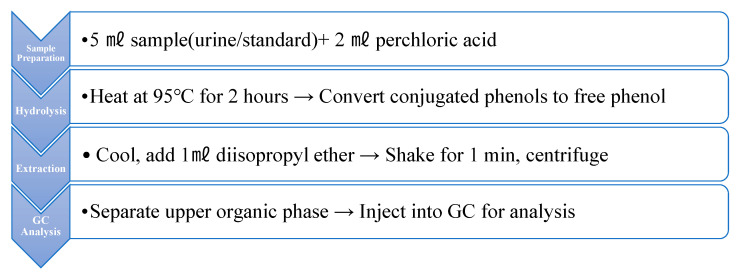
Analysis of phenol in urine.

The determination of urinary phenol was performed using gas chromatography (GC) under the conditions described in Table 3 [16]. The sample pretreatment process was conducted as illustrated in Figure 1.

Urinary creatinine levels were measured to adjust for urine dilution, and urinary phenol concentrations were expressed as mg/g creatinine. Creatinine adjustment is a widely accepted method in biological monitoring to normalize variations in urine concentration, providing a more accurate reflection of internal exposure dose [25].

While other biomarkers for phenol exposure exist, urinary phenol was chosen due to its established reliability as a direct metabolite of phenol and its common use in occupational exposure assessments [21]. We acknowledge that individual metabolic differences can influence urinary phenol levels, and this aspect was considered during data interpretation.

#### 2.2.5. Statistical Analysis

All collected data were analyzed using SPSS version 25.0 (IBM Corp., Armonk, NY, USA). Paired sample *t*-tests were conducted to evaluate the statistical significance of differences in urinary phenol concentrations before and after work. One-way analysis of variance (ANOVA) was performed to assess differences in urinary phenol levels according to drinking frequency, working hours, job type, frequency of skin exposure, and use of personal protective equipment (PPE). 

Furthermore, multiple linear regression analysis was applied to evaluate the independent effects of various occupational and lifestyle factors on urinary phenol concentrations. The operational definitions of the independent variables used in the regression model were as follows:

- Smoking status (categorical: current smoker, non-smoker/former smoker)
- Drinking frequency (categorical: once per week, twice per week, ≥3 times per week)
- Daily working hours (categorical: <2 h, 2–4 h, ≥4 h)
- Job type (categorical: patrol, sampling, cleaning)
- PPE use (categorical: used, not used)
- Frequency of skin exposure (categorical: once, twice, three times, ≥4 times per day)

A *p*-value of <0.05 was considered statistically significant.

## 3. Results

### 3.1. General Characteristics of Study Participants

A total of 41 male workers participated in this study, with a mean age of 40.7 years. The average total employment duration was 184.2 months (15 years and 3 months), and the average duration in the current job was 49.4 months (4 years and 1 month). The average daily working hours were 8 h (Table 4).

Among the participants, 90.2% (37 workers) reported consuming alcohol, while 34.1% (14 workers) were current smokers, and 65.9% (27 workers) were either non-smokers or former smokers. Regarding the use of respiratory protective equipment, 53.7% (22 workers) wore a gas mask, whereas 46.3% (19 workers) did not.

In addition to gas masks, the usage rate of additional skin protective equipment (e.g., gloves, goggles) was 19.5% (eight workers), while 80.5% (thirty-three workers) did not use any additional protective gear. Analysis of the frequency of skin exposure by work type revealed that exposure three times per day was the most common (53.7% of participants). Furthermore, the actual working time during an 8 h shift was most commonly between 4 and 5 h (43.9%) (Table 4).

### 3.2. Correlation Between Airborne Phenol and Post-Shift Urinary Phenol

The mean airborne phenol concentration was measured at 0.9051 ± 0.9336 mg/m^3^. The mean urinary phenol concentration was 100.27 ± 75.76 mg/g creatinine before work and 138.13 ± 109.58 mg/g creatinine after work.

**Figure 2:**
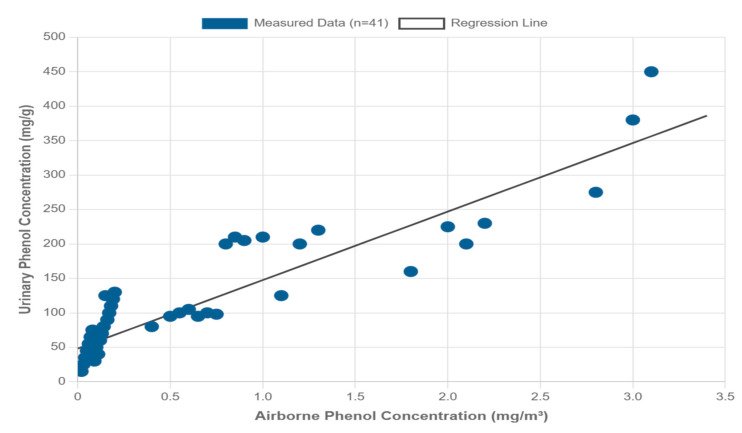
Correlation between airborne phenol concentration and urinary phenol levels (r = 0.8471, *p* = 0.03), indicating a strong exposure–response relationship.

Analysis of the correlation between airborne phenol concentration and creatinine-adjusted urinary phenol concentration among the exposed group revealed a statistically significant correlation (*p* = 0.03). The linear regression analysis produced the equation y = 99.439x + 48.135 (r = 0.8471) (Figure 2).

These results indicate that as airborne phenol concentration increases, urinary phenol concentration also tends to increase.

### 3.3. Changes in Urinary Phenol Concentration According to Lifestyle Habits

To analyze the effect of personal lifestyle habits on urinary phenol concentration, differences were examined based on smoking, medication use, coffee consumption during work, alcohol intake, and exercise habits.

Smokers exhibited significantly higher urinary phenol concentrations compared to non-smokers, and this difference was statistically significant. Additionally, urinary phenol concentrations showed a significant increasing trend with higher alcohol consumption frequency. In contrast, other lifestyle variables such as medication use, coffee consumption, and exercise habits did not show statistically significant differences in urinary phenol concentrations (Table 5).

### 3.4. Changes in Urinary Phenol Concentration According to Work Characteristics

#### 3.4.1. Differences in Urinary Phenol Concentration by Job Type

Analysis of urinary phenol concentrations according to job type revealed that sampling workers and cleaning workers exhibited approximately seven and eight times higher urinary phenol concentrations, respectively, compared to patrol workers. These results indicate that job types significantly influence urinary phenol concentrations (Table 6).

An analysis of urinary phenol concentration before and after work revealed a statistically significant increase in post-shift urinary phenol levels compared to pre-shift levels. The mean pre-shift urinary phenol concentration was 100.27 ± 75.76 mg/g creatinine, which increased to 138.13 ± 109.58 mg/g creatinine post-shift. Notably, cleaning workers exhibited a 1.6-fold increase in urinary phenol concentration before and after work, indicating a higher exposure level due to the nature of their tasks (Table 6).

#### 3.4.2. Differences in Urinary Phenol Concentration by Working Hours

Analysis of urinary phenol concentrations according to working hours showed a statistically significant increasing trend with longer working hours. In particular, workers who worked for more than 2 h exhibited over three times higher urinary phenol concentrations compared to those who worked less than 2 h, demonstrating a direct relationship between working hours and phenol accumulation (Table 7).

#### 3.4.3. Differences in Urinary Phenol Concentration by Skin Exposure Frequency

An analysis of the relationship between skin exposure frequency and urinary phenol concentration showed a significant increase in urinary phenol levels as skin exposure frequency increased. Notably, workers with four or more skin exposures exhibited more than three times higher urinary phenol concentrations compared to those with fewer than two exposures. While exposure less than twice did not show a significant impact, workers exposed two or more times exhibited a more than twofold increase in urinary phenol concentration, with a progressive increase observed as exposure frequency rose (Table 7).

#### 3.4.4. Differences in Urinary Phenol Concentration by Protective Equipment Use

Comparison by protective equipment indicated that workers who wore respiratory protection showed a mean urinary phenol concentration of 150.23 ± 98.33 mg/g creatinine, whereas non-users averaged 124.14 ± 122.57 mg/g creatinine; this difference was not statistically significant (*p* = 0.45). In contrast, skin protection exerted a clear effect: employees without skin PPE recorded markedly higher urinary phenol levels (156.65 ± 113.84 mg/g creatinine) than those wearing gloves or other barriers (61.76 ± 33.21 mg/g creatinine; *p* < 0.05) (Table 7). Experimental glove-permeation data support these findings double-layer or butyl-rubber gloves reduce phenol breakthrough time by roughly 50% [18], while, under simulated hand movement, disposable nitrile gloves exhibit more than a threefold increase in permeation rate [19].

### 3.5. Factors Influencing Urinary Phenol Concentration

A multiple regression analysis was conducted to determine the key factors affecting urinary phenol concentration. Model I included airborne phenol concentration as the only independent variable, while Model II incorporated work-related variables such as work duration, skin exposure frequency, job type, and workplace setting (indoor vs. outdoor). Finally, Model III added personal health characteristics, including smoking and alcohol consumption.

According to the analysis results, Model I showed that urinary phenol concentrations significantly increased with higher airborne phenol concentrations. In Model II, both airborne phenol concentration and job type significantly influenced urinary phenol concentrations. Model III identified airborne phenol concentration, working hours, skin exposure frequency, and smoking status as significant influencing factors. Specifically, smokers exhibited significantly higher urinary phenol concentrations compared to non-smokers, and urinary phenol concentrations were also significantly higher in cases of longer working hours or skin exposure occurring five or more times (Table 8).

## 4. Discussion

This study investigated the relationship between occupational exposure to airborne phenol and its biological accumulation, considering various occupational and lifestyle factors. Our findings provide valuable insights into the dynamics of phenol exposure in a real-world industrial setting and highlight critical areas for intervention. 

The statistically significant correlation observed between airborne phenol concentration and urinary phenol levels (r = 0.8471, *p* = 0.03) confirms that urinary phenol is a reliable biomarker for assessing occupational phenol exposure, even at low airborne concentrations. This finding is consistent with previous research demonstrating a direct relationship between environmental phenol levels and biological uptake [11,12]. The linear regression equation (y = 99.439x + 48.135) further quantifies this relationship, providing a predictive model for estimating internal exposure based on airborne concentrations. 

Our results indicate a significant increase in urinary phenol levels from pre-shift to post-shift, particularly among cleaning workers, who exhibited a 1.6-fold increase. This underscores the importance of task-specific exposure assessment and highlights that certain job roles carry a higher risk of phenol absorption. The higher exposure among cleaning workers is likely attributable to direct contact with phenol-containing materials during cleaning processes, emphasizing the need for stringent personal protective equipment (PPE) usage and adherence to safety protocols in such roles. 

The finding that smokers had significantly higher urinary phenol levels (194.54 ± 137.52 mg/g creatinine) compared to non-smokers (108.88 ± 80.10 mg/g creatinine, *p* = 0.046) is consistent with existing literature [5,14,15,16,17,18,19,20,21,22,23]. Tobacco smoke is a known source of phenol, and this result reinforces the notion that lifestyle factors significantly contribute to an individual’s overall phenol burden. This highlights the importance of comprehensive exposure assessments that consider both occupational and non-occupational sources, and it strongly supports the implementation of smoking cessation programs for workers in phenol-exposed environments. 

Interestingly, our study revealed that workers who reported using protective masks had higher urinary phenol levels (150.23 ± 98.3 mg/g creatinine) than those who did not (124.63 ± 119.2 mg/g creatinine, *p* = 0.002). This unexpected finding warrants further discussion. Several factors could contribute to this observation. Firstly, it is possible that workers who perceive themselves to be at higher risk, or who are engaged in tasks with inherently higher exposure potential, are more diligent in wearing masks. In such cases, despite mask usage, their overall exposure might still be higher due to the nature of their work. Secondly, the effectiveness of the masks used, their proper fit, and consistent usage throughout the work shift could be critical factors. Improperly fitted or used masks may not provide adequate protection, leading to continued exposure. Thirdly, this could suggest that dermal absorption plays a more significant role in overall phenol uptake than previously assumed, and mask usage alone is insufficient to mitigate exposure from other routes. Future research should investigate the specific types of masks used, their fit-testing protocols, and the relative contributions of inhalation versus dermal absorption in different work scenarios to fully understand this finding. The statistically significant increasing trend of urinary phenol concentrations with longer working hours further emphasizes the cumulative nature of phenol exposure. This suggests that prolonged exposure, even at low concentrations, can lead to a greater internal dose. This finding has important implications for work–rest schedules and exposure limits, indicating that time-weighted average limits alone may not fully capture the risk associated with extended work shifts.

This study has several limitations. Firstly, the sample size of 41 workers, while sufficient for statistical analysis, limits the generalizability of our findings to a broader population of phenol-exposed workers. Secondly, while we collected information on lifestyle habits, a more detailed assessment of dietary intake and other potential environmental sources of phenol could provide a more comprehensive understanding of total phenol exposure. Thirdly, the cross-sectional nature of this study limits our ability to establish causality between exposure and health outcomes. Longitudinal studies are needed to track changes in urinary phenol levels over time and correlate them with long-term health effects.

Despite these limitations, this study provides valuable insights into occupational phenol exposure and its determinants. Our findings underscore the importance of integrated exposure assessment strategies that consider both airborne concentrations and biological monitoring, along with individual lifestyle factors. The results highlight the need for continuous improvement in workplace ventilation, strict adherence to PPE protocols, and the promotion of healthy lifestyle choices among workers. Future research should focus on larger cohorts, incorporate more detailed exposure assessment methods, and explore the long-term health impacts of chronic low-dose phenol exposure.

## 5. Conclusions

This study provides robust evidence that occupational exposure to airborne phenol, even at relatively low concentrations, leads to measurable biological accumulation, as reflected in elevated urinary phenol levels. More importantly, the analysis reveals that internal dose cannot be fully explained by airborne exposure alone, underscoring the multifactorial nature of phenol accumulation in real-world industrial settings.

Occupational characteristics—particularly job role, frequency of dermal contact, and work duration—were shown to significantly affect urinary phenol levels, with cleaning workers exhibiting disproportionately higher burdens. This highlights the critical role of dermal absorption in phenol toxicokinetics, a pathway often underestimated in conventional, inhalation-focused assessments. Furthermore, lifestyle factors such as smoking and alcohol consumption were independently associated with elevated urinary phenol concentrations, reinforcing the need for an integrated model of occupational exposure that accounts for individual behavioral risks.

Clinically, this means that workers with elevated urinary phenol levels, even in the absence of overt symptoms, may be at an increased risk for long-term health complications, such as renal and hepatic impairment and potential neurotoxic effects. Therefore, our results advocate for a paradigm shift towards worker-centered health protection frameworks that include regular biological monitoring, targeted health education, and early clinical intervention to mitigate the potential for chronic phenol-related health issues and improve long-term worker well-being.

These findings have several important implications. First, biological monitoring should be institutionalized as a complementary measure to airborne sampling in phenol-exposed workplaces, providing a more accurate and individualized assessment of internal exposure. Second, occupational hygiene interventions must prioritize dermal protection strategies, particularly among high-risk job categories. Third, exposure mitigation policies should be expanded to incorporate health promotion initiatives, including behavior modification programs targeting smoking cessation and alcohol moderation.

From a regulatory and public health perspective, this study calls for a paradigm shift in exposure assessment and control—from reliance on environmental concentration thresholds to biologically meaningful indicators that reflect total body burden. This shift is essential for developing more effective, worker-centered health protection frameworks.

Finally, future research should pursue longitudinal and mechanistic studies to clarify the cumulative and chronic health effects of low-level phenol exposure. Incorporating genetic susceptibility and metabolic profiling may also enhance risk stratification. Simultaneously, interventional trials evaluating the effectiveness of engineering controls and targeted education programs would contribute valuable evidence for policy formulation.

## Figures and Tables

**Table 1:** BPA exposure assessment by task type.

Task Type	Task Description	BPA Exposure Potential
Patrol	Maintenance and inspection of process equipment	Low
Sampling	BPA quality evaluation and sample collection	Medium
Cleaning	Cleaning of pipelines and equipment	High

**Table 2:** HPLC conditions for airborne phenol analysis.

HPLC Chromatograph Condition	
Detector	UV 218 nm
Flow rate	1.0 mL/min
Injection volume	20 μL
Column	Alltima-C18
Mobile phase	ACN & KH2PO430:70

**Table 3:** Gas chromatograph condition for analyzing phenol in urine.

Gas Chromatograph Condition	
Detector	Gas Chromatograph with Flame-Ionization Detector
Column	Carbowax-20 (50 m)
Injection temp	200 °C
Detector temp	250 °C
Column temp	150 °C
Nitrogen flow rate	60 mL/min

**Table 4:** General characteristics for participants.

Variables		Mean ± S.D. (Range)	
Age		40.7 ± 6.4 (28.0~55.0)	
Years of employment		15.3 ± 1.6 (4.9~35)	
Years for current job		4.1 ± 2.7 (1.2~17.8)	
		Frequency (%)	
Drinking	Yes		37 (90.2)
	No		4 (9.8)
Smoking	Current		14 (34.1)
	Non- and ex-smoker		27 (65.9)
Mask	Using		22 (53.7)
	Non-using		19 (46.3)
Items for skin protection	Using		8 (19.5)
	Non-using		33 (80.5)
No. of skin exposures	1		4 (9.8)
	2		9 (22.0)
	3		22 (53.7)
	≥4		6 (14.6)
Work time	1~2		9 (22.0)
	2~3		6 (14.6)
	3~4		8 (19.5)
	4~5		18 (43.9)

**Table 5:** Relationship between phenol urine after working and general characteristics.

Variables		Mean ± S.D	*p*-Value
Smoking	Current smoking	194.54 ± 137.52	0.046
	Non- and ex-smoking	108.88 ± 80.10	
Drug	Yes	147.22 ± 79.20	0.864
	No	137.15 ± 113.18	
Coffee	Yes	111.16 ± 101.65	0.684
	No	127.56 ± 75.26	
Exercise	Yes	110.95 ± 98.51	0.712
	No	101.65 ± 100.33	
Weekly drinking	1	104.61 ± 91.27	0.045
	2	137.51 ± 124.72	
	≥3	177.18 ± 130.43	
		[Unit: mg/g creatinine]	

**Table 6:** Difference between phenol in urine before and after working by work type.

Work Type	N	Before Working (Mean ± S.D)	After Working (Mean ± S.D)	*p*-Value
Washing	17	104.19 ± 73.27	176.63 ± 109.78	0.002
Sampling	18	117.31 ± 76.59	138.39 ± 104.91	0.372
Patrol	06	23.91 ± 22.03	28.32 ± 24.17	0.457
Total	41	100.27 ± 75.76	138.14 ± 109.58	0.006
		[Unit: mg/g creatinine]		

**Table 7:** Difference between phenol in urine by work characteristics.

Variables		N	Mean ± S.D	*p*-Value
Daily working time (h)	<2	9	47.71 ± 36.37	0.006
	2~4	14	135.49 ± 105.85	
	≥4	18	185.40 ± 111.20	
Work type	Patrol	6	28.32 ± 24.16	0.020
	Sampling	18	138.39 ± 104.90	
	Washing	17	176.63 ± 109.77	
No skin exposure	<2	4	55.72 ± 17.14	0.039
	2~4	9	129.26 ± 134.46	
	≥4	28	152.76 ± 105.58	
Mask	Using	22	150.23 ± 98.33	0.454
	Non-using	19	124.14 ± 122.57	
Items for skin protection	Using	8	61.76 ± 33.212	0.032
	Non-using	33	156.65 ± 113.84	
		[Unit: mg/g creatinine]		

**Table 8:** Multiple regression for phenol in urine.

Variables		Model I		Model II		Model III	
		Regression Coefficient	*p*-Value	Regression Coefficient	*p*-Value	Regression Coefficient	*p*-Value
Phenol concentration in air		99.44	<0.0001	108.48	<0.0001	110.89	<0.0001
Mask				−13.39	0.56	−6.68	0.78
Items for skin protection				24.29	0.37	22.55	0.45
Working area				−48.38	0.17	−52.33	0.15
Working time (h)	<2			1.00	-	1.00	-
	2~4			56.42	0.16	98.00	0.01
	≥4			67.08	0.08	103.79	0.01
No. of skin exposures	<4			1.00	-	1.00	-
	4~5			−13.78	0.59	−19.70	0.45
	≥5			17.95	0.62	90.74	0.02
Work type	Washing			1.00		1.00	
	Sampling			−17.56	0.46	6.51	0.78
	Patrol			−2.51	0.94	−18.45	0.57
Smoking						102.60	0.04
Drinking	Non-drinking					1.00	-
	1/week					15.38	0.73
	2/week					6.43	0.89
	≥3/week					−33.68	0.52
Intercept			48.14		55.05		25.26
Adj.R 2			0.71		0.71		0.78
R 2			0.72		0.78		0.86
F-value			99.15		10.88		10.24
*p*-value			<0.0001		<0.0001		<0.0001

Model I: adjusted for phenol concentration in air; Model II: adjusted for model I + work characteristics; Model III: adjusted for model II + general characteristics.

## Data Availability

The data presented in this study are available on request from the corresponding author.
